# Therapeutic Targeting the Cell Division Cycle 25 (CDC25) Phosphatases in Human Acute Myeloid Leukemia — The Possibility to Target Several Kinases through Inhibition of the Various CDC25 Isoforms 

**DOI:** 10.3390/molecules191118414

**Published:** 2014-11-12

**Authors:** Annette K. Brenner, Håkon Reikvam, Antonio Lavecchia, Øystein Bruserud

**Affiliations:** 1 Section for Hematology, Institute of Clinical Science, Faculty of Medicine and Dentistry, University of Bergen, Bergen, 5021, Norway; 2 Department of Medicine, Haukeland University Hospital, Bergen 5021, Norway; 3 “Drug Discovery” Laboratory, Department of Pharmacy, University of Naples Federico II, Naples 80131, Italy

**Keywords:** CDC25, cell cycle, kinase, CDC25 inhibitors, anticancer agents, AML

## Abstract

The cell division cycle 25 (CDC25) phosphatases include CDC25A, CDC25B and CDC25C. These three molecules are important regulators of several steps in the cell cycle, including the activation of various cyclin-dependent kinases (CDKs). CDC25s seem to have a role in the development of several human malignancies, including acute myeloid leukemia (AML); and CDC25 inhibition is therefore considered as a possible anticancer strategy. Firstly, upregulation of CDC25A can enhance cell proliferation and the expression seems to be controlled through PI3K-Akt-mTOR signaling, a pathway possibly mediating chemoresistance in human AML. Loss of CDC25A is also important for the cell cycle arrest caused by differentiation induction of malignant hematopoietic cells. Secondly, high CDC25B expression is associated with resistance against the antiproliferative effect of PI3K-Akt-mTOR inhibitors in primary human AML cells, and inhibition of this isoform seems to reduce AML cell line proliferation through effects on NFκB and p300. Finally, CDC25C seems important for the phenotype of AML cells at least for a subset of patients. Many of the identified CDC25 inhibitors show cross-reactivity among the three CDC25 isoforms. Thus, by using such cross-reactive inhibitors it may become possible to inhibit several molecular events in the regulation of cell cycle progression and even cytoplasmic signaling, including activation of several CDKs, through the use of a single drug. Such combined strategies will probably be an advantage in human cancer treatment.

## 1. Introduction

Reversible protein phosphorylation is an important molecular mechanism for the regulation of protein activity; this is true for many proteins that are essential for the regulation of important cellular events like intracellular signaling, cell cycle progression and proliferation, cellular differentiation and induction of apoptosis [1]. Protein phosphorylation and dephosphorylation are catalyzed by protein kinases and protein phosphatases, respectively [1]. The protein phosphatases can be divided into two different subclasses; the first class specifically hydrolyses serine/threonine phosphoesters whereas the second class is referred to as tyrosine phosphatases and specifically hydrolyses phosphotyrosine [2]. The tyrosine phosphatases also include a family of dual specificity phosphatases that hydrolyze both phosphotyrosines and serine/threonine phosphoesters; the cell division cycle 25 (CDC25) phosphatases are a subset among these dual specificity phosphatases [2]. This family includes CDC25A, CDC25B and CDC25C [3]; these three enzymes are important regulators of several steps in the cell cycle and they possibly have a role in the development of a variety of human malignancies [2,4]. CDC25 inhibition is therefore considered as a possible therapeutic strategy in cancer treatment.

Cyclin-dependent kinases (CDKs) are important regulators of cell cycle progression, and CDK inhibition can be accomplished through inhibition of activating phosphatases [2]. Several CDKs are activated by CDC25A/B/C, and CDK inhibition can therefore be achieved through CDC25 inhibition [2,5,6]. However, the CDC25s also have additional functions in cell cycle regulation, and several CDC25 inhibitors show cross-reactivity among the three CDC25 forms [2,5,6]. Thus, by using cross-reactive CDC25 inhibitors it will be possible to target several steps in cell cycle regulation, including several cyclin-dependent kinases, through the use of a single therapeutic agent.

## 2. CDC25 and the Regulation of Cell Cycle Progression

### 2.1. The Structure of CDC25

**Figure 1:**
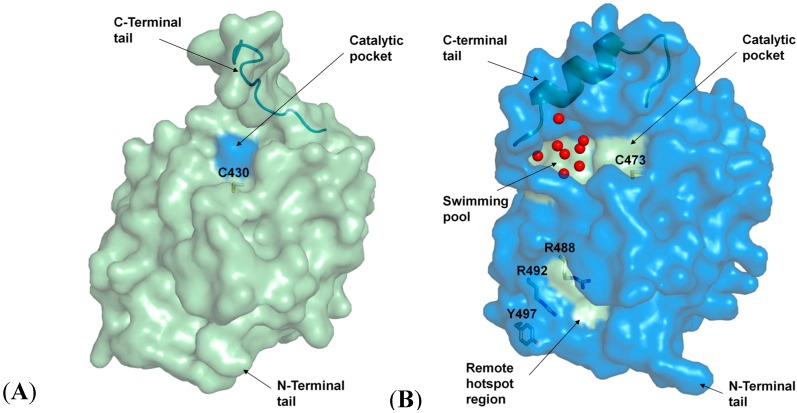
Structures and domains of CDC25A and CDC25B. (**A**) Surface view of CDC25A (palegreen). The catalytic site is indicated in blue marine. The C-terminal tail (residues 484-495) is shown in palegreen cartoon. (**B**) Surface view of CDC25B (blue marine). The catalytic site and the adjacent “swimming pool” pocket are indicated in palegreen. The water molecules are represented as red spheres. The hotspot residues (R488, R492 and Y497), which govern the association with the protein substrate, are shown in blue sticks. The remote hotspot region is highlighted in palegreen. The α-helical C-terminal tail (residues 531–550) is shown in palegreen cartoon.

CDC25 phosphatases are conserved eukaryotic proteins [7]. The three different human isoforms CDC25A/B/C contain 524, 580 and 473 amino acids, respectively, resulting in molecular masses in the 53–65 kDa range [8]. The proteins consist of two domains: a highly conserved catalytic domain at the C-terminal and an N-terminal regularity region. The catalytic domain contains the HCX_5_R motif typically of tyrosine phosphatases [9]. The dipole moment of an adjacent α-helix favors the deprotonation of the catalytic cysteine increasing its reactivity towards phosphorylated threonine and tyrosine residues [10]. According to the crystal structures of human CDC25A and CDC25B [11,12], the isoforms differ in their amino acid arrangements around the catalytic site which supposedly is linked to divergences in specificity and activity [13]. As depicted in Figure 1, the two structures have near identical folding in all conserved regions, with the only significant degeneration occurring in the extreme C-termini. Whereas the CDC25B C-terminal tail (residues 531–550) folds upon itself, perhaps to aid in substrate binding, the CDC25A tail (residues 484–495) is directed away from the active site, leaving it largely exposed. In contrast to other phosphatases, the active site of CDC25s is flat and shallow, suggesting a broad interaction interface. One of the largest cavities on the surface of CDC25B is adjacent to the catalytic pocket and is called the “swimming pool” because of the abundance of well-ordered water molecules it contains [14]. It seems as if substrate recognition relies on so-called hotspot residues (R488, R492 and Y497 on CDC25B) 20–30 Å apart from the active site [15]. Recent identification of a hotspot interaction between CDC25B and its native substrate, the CDK2/cyclin A complex, has moved some drug discovery attempts targeting CDC25B from the catalytic pocket to the remote hotspot region [16]. The regulatory domain at the N-terminal, on the other hand, contains several phosphorylation sites which regulate the activity and stability of the phosphatases, and their interaction with other proteins [17]. The domain also has signal peptides (nuclear localization sequence, NLS, and nuclear exportation sequence, NES) that determine the intracellular localization of the CDC25 proteins [18,19].

### 2.2. CDC25 in Cell Cycle Regulation

**Figure 2:**
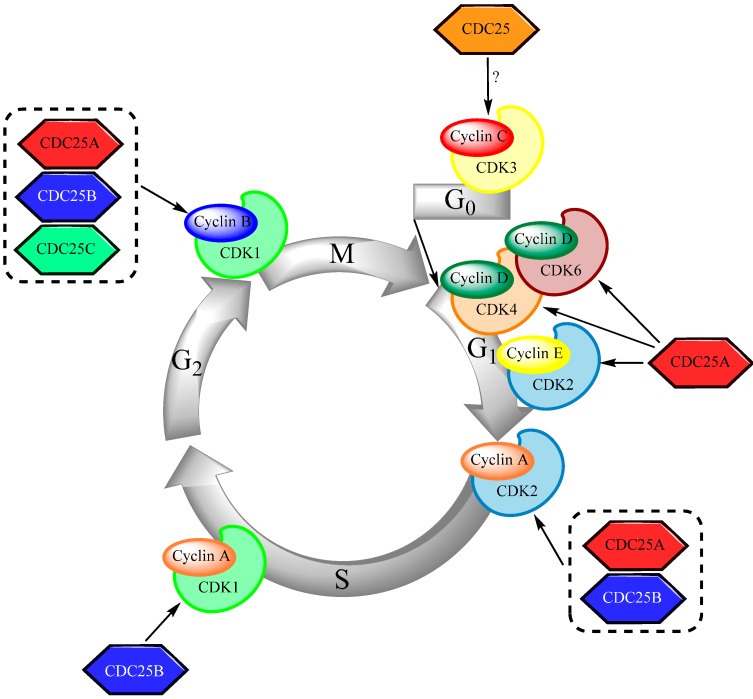
An overview of the regulatory function of CDC25s in cell cycle progression. At G_0_-phase, cells that had been quiescent, re-enter the cell cycle after activation of CDK3/cyclin C. Dephosphorylation of CDK4/cyclin D, CDK6/cyclin D and CDK2 both in complex with cyclin E and cyclin A by CDC25s leads to the transition into the DNA-replication phase. At late S-phase, CDC25B activates CDK1/cyclin A. Finally, dephosphorylation of CDK1/cyclin B triggers mitotic entry, and in this important step all three CDC25 isoforms are involved. At the end of mitosis, both CDK1/cyclin B and the CDC25s are degraded and the cycle can start all over again.

CDC25 phosphatases function as cell cycle regulators: they activate CDKs by dephosphorylating two residues within the ATP-binding loop [20]. The CDC25 isoforms fulfil different tasks throughout the cell cycle; this is also demonstrated by their localization as CDC25A predominantly is a nuclear protein [21] whereas the other two isoforms shuttle in and out the nucleus throughout cell cycle progression [22,23]. A simplified overview of the cell cycle regulation is shown in Figure 2. It is believed that all three isoforms are essential for the proper execution of the cell cycle, although it has been shown that CDC25A alone is sufficient for initiation of each step in the cycle [23,24].

The CDK3/cyclin C complex initiates the re-entry of quiescent cells into the cell cycle from G_0_-phase. In middle G_1_-phase, CDC25A activates CDK4 and CDK6 that are in complex with cyclin D [25]. The latter at least partially inhibits retinoblastoma protein (Rb) in the G_1_/S-transition rate-limiting step [26], thus permitting transcription of genes that are required for cell cycle progression, including CDC25A and other cyclins [27,28]. CDC25A overexpression accelerates the entry into the DNA replication phase by dephosphorylation of CDK2/cyclin E, which additionally phosphorylates Rb [25] and mutually activates CDC25A in a feed-forward loop [21,29]. CDC25A reaches its highest expression level at late G_1_-phase, where it together with CDC25B facilitates the transition into S-phase by dephosphorylation of CDK2/cyclin A, which in turn activates DNA replicating proteins [30]. CDC25C controls the initiation of the S-phase [31]. At late S-phase, CDC25B dephosphorylates CDK1/cyclin A, a less active complex compared to CDK2/cyclin A, which persists throughout G_2_-phase [30]. Dephosphorylation of CDK1/cyclin B by CDC25 is the rate-limiting step for the G_2_ to mitosis transition [32]. Active CDK1/cyclin B, that is inhibited by the Wee1 and Myt1 kinases [33], is the main trigger for mitosis. When CDK1 reaches a critical concentration level, it gets dephosphorylated by CDC25. At the same time, activated CDK1/cyclin B inactivates its inhibitor Wee1, thus resulting in an abrupt transition into mitosis [34]. All three CDC25 isoforms are involved in the G_2_/M-transition but CDC25B appears to take the key role in this process [35]. CDC25B is most abundant and active at late G_2_-phase and during mitosis, and is localized to the centrosome where it dephosphorylates CDK1/cyclin B [35,36]. The latter is subsequently translocated to the nucleus, where it is completely activated by CDC25C leading to mitotic onset [30]. CBC25B is then phosphorylated and this is supposed to be important for the regulation of the mitotic entry [37]. At the end of mitosis, both CDK1/cyclin B and the CDC25s are degraded resulting in transition into interphase. The proteins are subjected to anaphase promoting complex/cyclosome (APC/C)-dependent ubiquitination leading to proteasome-mediated degradation [38]. It also seems as if CDC25s can be inactivated by CDC14 family members that dephosphorylate the residues that previously had been phosphorylated by CDK1 [39].

Results from mice lacking CDC25B [40], CDC25C [22] or both [23] indicate that these proteins are not essential for cell cycle progression or for control of checkpoint responses. Additionally, concentration levels of CDC25A were not significantly altered in these models, suggesting that the third isoform does not compensate for the loss of the two others [23].

### 2.3. Control of CDC25 Expression and Activity

**Figure 3:**
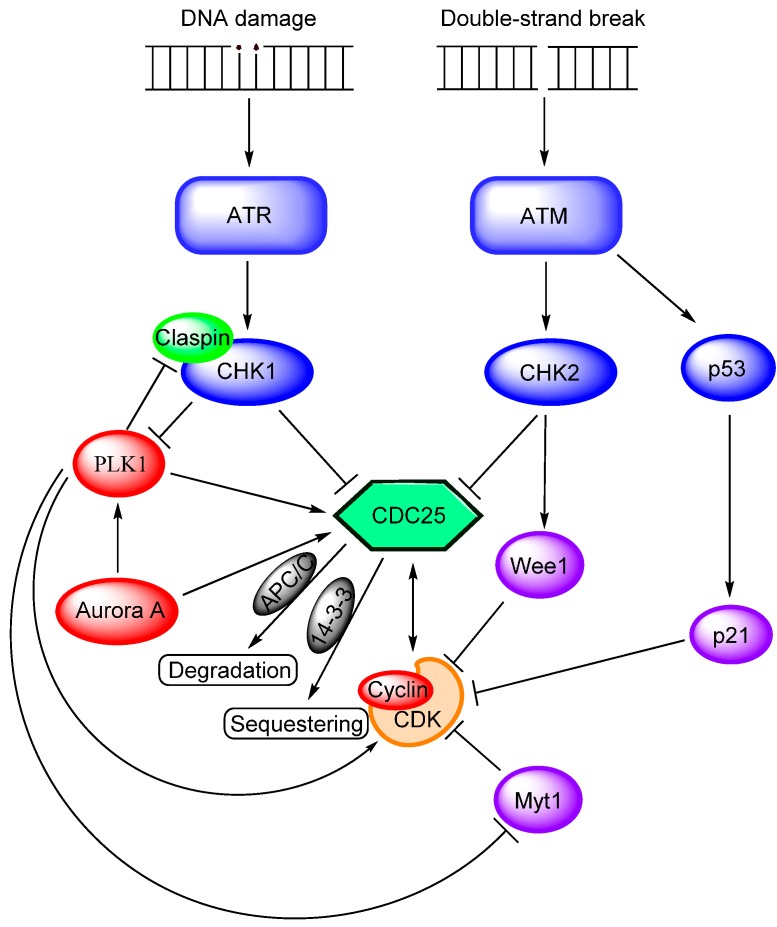
Molecular interactions that regulate CDC25 activity (for simplicity reasons the effects of PI3K-Akt-mTOR signaling are described in the text but not included in the figure). The CDC25 activators are shown in red, the upstream inhibitors in blue and the downstream regulators in purple. Note that the CDC25s and the CDKs mutually activate each other. PLK1 is a key component as it positively regulates CDC25s and two of their activators, as well as it inhibits Myt1 and mediates the degradation of claspin. The key components for down-regulation of CDC25s are ATR and ATM. CDC25s are also prone to degradation by APC/C-dependent ubiquitination and nuclear exclusion by 14-3-3 binding. See text for further description.

Because the CDC25s are essential for regulation of the cell cycle, their expression needs to be highly controlled. Dysregulation in the case of CDC25A, for example, accelerates the G_1_/S-phase transition [41], whereas an overexpression of CDC25B leads to premature mitotic entry [42]. The phosphatases are regulated both prior to transcription and post-translationally. At the transcription level, CDC25s are activated by the transcription factors E2F1, E2F2 and E2F3—the antagonists of Rb. Signal transducer and activator of transcription 3 (STAT3) usually activates *CDC25* gene transcription, but through Rb recruitment it can also exhibit an inhibitory effect [43]. At the post-translational level, CDC25s are subject to protein modifications, both ubiquitination prior to degradation (described above) and phosphorylation. The latter is directed to mainly serines positioned in the N-terminal regulatory domain. Phosphorylation can either activate or inhibit the CDC25 phosphatases, leading to alterations in their catalytic activity, subcellular localization, substrate recognition and stability [17]. CDKs are the most important activators: CDK1/cyclin B mutually activates both CDC25B and CDC25C in a feed-forward loop resulting in mitotic entry, whereas CDK2/cyclin E and CDC25A form another feed-forward loop leading to DNA replication onset. Two other important kinases positively regulate CDC25s and promote mitosis: the polo-like kinase 1 (PLK1) and Aurora kinases. The former activates CDC25C both directly and indirectly by CDK1/cyclin B phosphorylation and inhibition of the Wee1-like kinase Myt1 [44], in addition to favoring the nuclear import of CDC25C [30,45], whereas the latter activates both PLK1 and CDC25s [46,47]. PLK1 also plays a role in mitotic exit, as it is a positive regulator of the APC/C activity [48]. An overview of the most important activation and inhibition pathways is shown in Figure 3.

### 2.4. Cell Cycle Arrest and CDC25 Inhibition

Cell cycle progression can be arrested at three stages: before entry into S-phase, during S-phase and prior to mitosis. At the G_1_/S checkpoint, DNA synthesis is inhibited, whereas intra-S phase arrest blocks mitotic entry until the S-phase is completed [30]. Finally, at the G_2_/M checkpoint, damaged cells are arrested in order to allow for cell repair or apoptosis [49]. CDC25s are inactivated by checkpoint kinases (CHK1 and CHK2) in an ataxia-telangiectasia mutated (ATM) and AT and Rad3-related (ATR) kinases-dependent manner. Upon DNA single-strand damage, ATR activates CHK1, whereas ATM activates CHK2 and the tumor suppressor protein p53 mainly as a result of double-strand breaks [45,50]. Activated CHK1/CHK2 target CDC25 leading to its inhibition or degradation. The checkpoint kinases also increase the amount of Wee1 resulting in inactivation of CDKs [50], and the CDC25 activator PLK1 appears to be inhibited in an ATM/ATR-CHK1/CHK2-dependent manner. In detail, CHK2 inhibits CDC25A through p53 [51] resulting in inactivation of CDK4/cyclin D and CDK2/cyclin E, thus blocking S-phase entry [51,52]. On the other hand, all three isoforms of CDC25 are phosphorylated by CHK1 in order to prevent mitotic onset. Phosphorylated CDC25A/B can no longer activate CDK1/cyclin B [53,54], and inactivation of CDC25B/C sequesters the proteins in the cytoplasm [37,55]. Also, hyperphosphorylation of CDC25A leads to its degradation [53,56]. The checkpoints are silenced after repair or degradation of the damaged cells [49], and the re-entry into mitosis upon DNA-damage arrest is controlled by CDC25B upon activation by PLK1 [57]. PLK1 also inactivates CHK1 by mediated degradation of Claspin, the adaptor and activating partner of CHK1 [58].

In addition to the checkpoint kinases, several other proteins are involved in CDC25 inhibition, for example protein kinase B (PKB/Akt) and mitogen-activated protein kinases (MAPKs). The latter negatively regulate CDC25 upon DNA damage mediated by heat shock, oxidative stress, irradiation, feed deprivation and chemotherapy [30,59]. The effects of the PI3K-Akt-mTOR pathway on cell cycle progression are not completely understood and conflicting results have been obtained. On one side, Akt sequesters CDC25s in the cytoplasm upon binding to protein 14-3-3, thus inhibiting mitosis [60]. Also, it seems as if CDC25B activity itself is required for the activation of Akt and the p38 MAPK kinase [61], thus indicating a mutual regulation of these proteins. On the contrary, several studies have shown that Akt acts as an initiator of mitosis [62,63] and inactivation of both CHK1 [64] and CHK2 [65] by Akt have been observed, thus circumventing the degradation of CDC25s. Following DNA damage for instance, Akt impairs the activation of CHK1 in an ATR-independent manner, thus circumventing the cell cycle checkpoint and inhibiting apoptosis [66]. In concordance with this, inhibition of Akt led to restored CHK1 activity [67].

## 3. Small Molecule CDC25 Inhibitors

**Figure 4:**
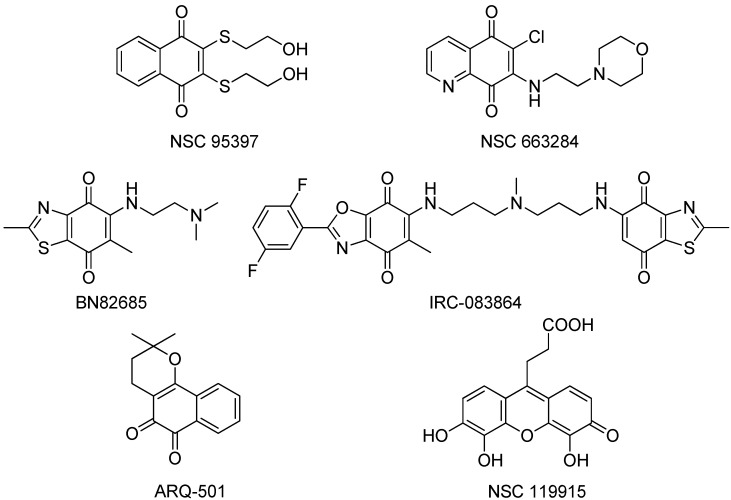
Structures of the most active quinone-containing inhibitors of CDC25 phosphatases.

Over the past few years, several synthetic and natural molecules with different structural features targeting CDC25 activity have been reported. Reviews by Lavecchia *et al.* provide a comprehensive overview of the current CDC25 inhibitor development [2,5,68,69]. The majority of the known CDC25 inhibitors belong to various chemical classes including phosphate bioisosteres, electrophilic entities, and quinonoids. These compounds act via reversible inhibition with the active site of CDC25s [70,71,72,73], irreversible inhibition of CDC25s by electrophilic modification [74,75] or oxidation of the critical cysteine residue in the catalytic domain (HCX_5_R) by reactive oxygen species (ROS) generated in cultured cells treated with quinone derivatives [76,77]. This latter mechanism could be consistent with the non-selective inhibition of CDC25 isoforms by quinone-type inhibitors. Moreover, ROS may oxidize other phosphatases, as well as unrelated cysteine-based enzymes, and therefore quinone-containing agents could potentially trigger a range of unrelated events in cells. To date, many of the most potent CDC25 inhibitors are quinone-containing compounds which inhibit all three isoforms of CDC25 in an unselective manner (Figure 4).

- NSC95397, an unspecific naphthoquinone isoform shows IC_50_ values in the mid nM range [72], significantly inhibits the proliferation of human and murine carcinoma cells and blocks the G_2_/M phase transition of the cell cycle.
- NSC 663284 is a potent, cell-permeable, and unspecific irreversible CDC25 inhibitor, arrests cells in the G_1_ and G_2_/M phases and induces significant growth inhibition of human breast cancer cell lines [75].
- The quinone-based compound BN 82685 inhibits all three mammalian CDC25 phosphatases in the high nM range in biochemical assays (IC_50_ = 250, 250, and 170 nM, respectively) and blocks the growth of human pancreatic tumor Mia PaCa-2 cells xenografted into athymic nude mice [78]. BN 82685 was further reported to retain its activity when taken orally. Furthermore, combining low concentrations of BN 82685 and paclitaxel (Taxol^®^), inhibits the proliferation of colon cancer cells [79], suggesting that combinations of CDC25 inhibitors with microtubule-targeting agents may be of therapeutic interest.
- The bis-quinonoid IRC-083864 is the most potent CDC25 inhibitor described thus far with an IC_50_ value of ~20 nM. It has activity in pancreatic and prostate cancer xenografts [80] and has entered into clinical trial under the name of Debio 0931, but no data are available yet. The two quinone groups of this large molecule are thought to deactivate the enzyme by covalent bond formation or oxidation of the critical active site thiolate anion.
- Another CDC25 inhibitor, ARQ-501, entered phase I clinical trials in patients with advanced and chemotherapy-unresponsive solid tumors, and is undergoing a phase II trial in patients with leiomyosarcoma and head and neck cancer, having completed an additional trial in combination with the nucleoside analog gemcitabine [81,82]. It is probable, however, that ARQ-501 is not directly a CDC25 inhibitor, but rather functions by some other mechanism.
- Recently, Lavecchia *et al.* discovered a new quinonoid CDC25 inhibitor (NSC 119915) with micromolar activity by means of a structure-based high-throughput virtual screening [24]. Mechanistically, NSC 119915 displays irreversible inhibition kinetics with *in vitro* K_i_ values for CDC25A and CDC25B of 70 and 80 nM, respectively, generates intracellular ROS in cells, arrests cells in the G_0_/G_1_ and G_2_/M phase transitions of the cell cycle, and significantly suppresses the growth of human MCF-7 breast, PC-3 prostate, and K562 leukemia cancer cell lines.

## 4. Biological Functions of CDC25 in Human AML

Relatively few studies have investigated the role of CDC25 in human AML. The most important studies are presented in detail in Table 1 [49,83,84,85,86,87,88,89,90,91], but it should be emphasized that several of these studies are relatively small, describe observations mainly in AML cell lines or used methodological strategies that would not be regarded as optimal today. Thus, knowledge about CDC25 in human AML is in our opinion incomplete, but despite this CDC25 should be regarded as a possible therapeutic target in human AML. The following main observations have been made in the available studies with regard to the various CDC25 isoforms in human AML (Table 1).

**Table 1:** The importance of CDC25 in AML; a summary of previous studies.

CDC25 Isoform [Reference] Experimental Model	Observations (Scientific Background, Observations, Conclusion)
**CDC25A** [83] AML cell lines (KBM3/Bu250 and OCI-AML3)	**Background:** The combination of fludarabine, clofarabine and busulfan has a cytotoxic effect on AML cells; the authors investigated the effect of adding the HDAC inhibitor suberoylanilide hydroxamic acid (SAHA) to the triple combination.
	**Observations:**
	1. HDAC inhibition increased the *antiproliferative* effect together with *caspase activation* and induction of *apoptosis*.
	2. Additional HDAC inhibition induced an ATM-initiated *DNA damage response* with activation of p53 and CHK2.
	3. The four-drug combination activated the ATM-CHK2-CDC25-CDK1 pathway with increased CHK2 phosphorylation, decreased CDC25A levels and thereby increased phosphorylation and deactivation of the downstream target CDK1.
	4. Similar observations could be seen in primary human AML cells (only 3 patients examined).
	**Conclusion:** Decreased CDC25A expression is seen in AML cells following exposure to antiproliferative and cytotoxic treatment.
**CDC25A** [84] Various cell lines, including the HL60 AML cell line	**Background:** The authors investigated the antiproliferative effects of extracts from the plant *Acalypha alopecuroidea.*
	**Observations:**
	1. The extract had an antiproliferative and cytotoxic effect.
	2. A DNA damage response was seen with CHK2 phosphorylation/activation subsequently leading to inactivation of CDC25A through phosphorylation and finally CDK1 Y15 hyperphosphorylation with cell cycle arrest.
	**Conclusion:** Antiproliferative and cytotoxic effects with cell cycle arrest are associated with CDC25A inactivation.
**CDC25A** [85] HL60 AML cells	**Background:** The anticancer-effect of extracts from the plant *Scutellaria orientalis* was investigated.
	**Observations:**
	1. The extract had a dose-dependent proapoptotic effect and induced genotoxic stress.
	2. Dose-dependent p21 induction together with dose-dependent CDC25A/cyclin D1 downregulation was also observed.
	**Conclusion:** Genotoxic stress and antiproliferative/proapoptotic effects are associated with downregulation of CDC25A.
**CDC25A** [86] Various cell lines, including AML lines	**Background:** The PI3K-Akt-mTOR pathway can be constitutively activated in human AML cells; the authors investigated a possible functional link between this pathway and CDC25A expression.
	**Observations: **
	1. Alk-dependent proliferation was inhibited by RNA interference-mediated downregulation of CDC25A.
	2. Pharmacological CDC25 inhibition reduced ALK (anaplastic lymphoma kinase)-dependent proliferation.
	3. PI3K-Akt-CDC25 mediated intracellular signaling downstream to the FLT3 growth factor receptor.
	**Conclusion:** High CDC25 protein levels are important for PI3K-Akt-mediated growth enhancement.
**CDC25A** [87] AML cell lines (KG1a, U937)	**Background:** Interactions with the microenvironment are important for regulation of proliferation for many different cells.
	**Observations:**
	1. Adhesion to fibronectin increased AML cell proliferation through increased S-phase entry; differentiated normal progenitors showed a similar effect whereas normal CD34^+^ cells showed decreased proliferation.
	2. The AML cells showed accumulation of CDC25A; pharmacological inhibition or siRNA-mediated downregulation of CDC25A impaired adhesion-dependent proliferation. CDC25A accumulation was CDH1 dependent and due to modified proteasomal degradation.
	3. The adhesion-induced proliferation and CDC25A upregulation was mediated through activation of the PI3K-Akt-mTOR pathway.
	**Conclusion:** CDC25A upregulation can be mediated by integrin-initiated signaling through PI3K-Akt-mTOR.
**CDC25B** [88] AML cell lines but also an immature subset of primary human AML cells (CD34^+^, high aldehyde dehydrogenase activity)	**Background:** The immediate-early response gene 5 (IER5) can function as a regulator of cell proliferation in cancer cells; the authors investigated whether IER5 is a regulator of AML cell proliferation.
	**Observations:**
	1. IER5 was constitutively expressed in human AML cells.
	2. Overexpression of IER5 caused a decrease in CDC25B associated with an antiproliferative effect and G_2_/M arrest.
	3. Downregulation of CDC25B mRNA expression was caused by IER5 binding to the promoter and mediated through NF-YB and p300.
	**Conclusion:** IER5-induced growth inhibition of immature AML cells is associated with downregulation of CDC25 expression.
**CDC25B** [49] AML cell lines (KG1a, U937) and primary human AML cells	**Background:** The importance of the G_2_/M checkpoint for the sensitivity of AML cells to genotoxic stress was examined; exposure to etoposide was used as the genotoxic stress.
	**Observations:**
	1. G_2_/M checkpoint stringency varied between AML cell lines; U937 cells showed an earlier exit from the checkpoint than KG1a cells after exposure to etoposide. Etoposide caused increased phosphorylation/activation of CHK1 in both cell lines, but the earlier exit by U937 cells was associated with an earlier decrease in CHK1 activation.
	2. CDC25B protein levels increased after etoposide exposure. CDC25B is important for checkpoint recovery, and pharmacological as well as siRNA inhibition of CDC25B caused an inhibition of the checkpoint recovery and entry into mitosis.
	3. Combination of etoposide + CHK1 inhibition increased the number of mitotic and apoptotic cells. Increased cytotoxicity was also seen for primary AML cells when combining etoposide with CHK1 inhibition, but this potentiating effect differed between patients.
	**Conclusion:** CDC25 is important for the sensitivity of human AML cells for genotoxic stress.
**CDC25B** [89] Primary human AML cells	**Background:** Is CDC25 important for the antiproliferative effect of PI3K/mTOR inhibitors in primary human AML cells?
	**Observations:**
	1. Resistance to PI3K/mTOR inhibition was associated with increased expression of CDC25B.
	2. Pharmacological CDC25 inhibition has an additive antiproliferative effect to PI3K/mTOR inhibition only for certain cell lines and for a subset of patients.
	**Conclusion:** The effect of CDC25 inhibition differs between patients, additive antiproliferative effects to PI3K-mTOR inhibition are seen only for a subset.
**CDC25C** [90] AML cell line U937	**Background:** Does CDC25C associate with cyclin A in human AML cells?
	**Observations: **
	CDC25C coprecipitated with cyclin A in proliferating cells, this was not seen after apoptosis induction through the extrinsic and intrinsic pathways.
	**Conclusion:** CDC25C contribute to cell cycle regulation in human AML cell lines through complexing with cyclin A.
**CDC25C** [91] Primary human AML cells	**Background:** Normal cells show different CDC25C splice variants, is this also true for primary human AML cells?
	**Observations: **
	Several splice variants were detected in primary human AML cells, and the splicing pattern differed between AML cells and normal CD34^+^ hematopoietic cells.
	**Conclusion:** Aberrant splicing may contribute to the AML cell phenotype and to functional differences in cell cycle regulation between patients.

CDC25 inhibitors can inhibit AML cell proliferation induced by integrin adhesion to fibronectin [87]; this integrin-initiated growth enhancement seems to be at least partly mediated through activation of PI3K-Akt-mTOR signaling, and its inhibition is thus an additional observation suggesting a functional interaction between CDC25 and PI3K/Akt/mTOR. The importance of CDC25 for Akt-dependent and integrin-initiated proliferation was described for the CDC25A isoform, whereas the other isoforms were not examined. However, most CDC25 inhibitors are not specific for CDC25 isoforms, *i.e.*, there are only quantitative but not qualitative differences in their activity with regard to inhibition of CDC25A/B/C [2] and it cannot be excluded that other isoforms and not only CDC25A contribute to the inhibition of Akt-dependent proliferation caused by pharmacological CDC25 inhibition. To conclude, our knowledge about CDC25 in human AML is incomplete and fragmentary but the observations so far suggest that there are functional interactions with both Aurora kinases, PLK1, NFκB and PI3K/Akt/mTOR; all these mediators are regarded as important for AML (stem) cell proliferation and survival. For this reason the possible role of CDC25s as potential therapeutic targets in human AML should be further investigated.

- *CDC25A.* Upregulation of CDC25A enhances lymphoma cell proliferation [87]. Its expression seems to be controlled through PI3K-Akt-mTOR signaling [87]. Loss of CDC25A is also important for cell cycle arrest during the differentiation of malignant hematopoietic cells [92]. This isoform seems to be important for the DNA damage response during exposure of human AML cells to cytotoxic drugs. It also seems important for Akt-dependent proliferation in AML cells because inhibition or knock-down of CDC25A reduces the Akt-dependent AML cell proliferation. The FLT3 gene is frequently mutated and thereby encodes a constitutively activated kinase in human AML and is an adverse prognostic factor [93]; this FLT3-initiated signaling is mediated through different pathways (including PI3K-Akt-mTOR) and CDC25 may thereby mediate or contribute to the adverse prognostic impact of this genetic abnormality.
- *CDC25B.* High expression is associated with resistance against the antiproliferative effect of PI3K-Akt-mTOR inhibitors [89], but the molecular mechanisms behind this effect are not known. Inhibition of this isoform seems to reduce AML cell line proliferation through effects on NF-YB and p300 [88]; whereas in other cells growth inhibition is seen only with combined Cyclin D1 and CDC25B inhibition [84]. These regulatory events thus represent a link between CDC25 and the NF-YB system that consist of the three subunits NF-YA, NF-YB and NF-YC; this system seems to contribute to carcinogenesis for several human malignancies [94]. Finally, activation of the NFκB system is also important for transcriptional regulation, and inhibition of NFκB at G_2_/M phase transition delays mitotic entry and inhibits transcription of G_2_/M-specific genes, including cyclin B, PLK1, and CDC25B [95]. NFκB is important for leukemic stem cell functions and their chemosensitivity [96], but it is not known whether this importance of NFκB on AML stem cells is mediated through its effects on CDC25B transcription.
- *CDC25C.* The molecule is encoded on chromosome 5 and CDC25C seems important for the phenotype of AML cells with chromosomal deletions involving this chromosome [97]. Malignant myeloid cells seem to express a splicing/isoform profile of CDC25C that is different from normal hematopoietic cells [91].

## 5. The Molecular Context of CDC25 in Human AML Cells

Cell cycle progression is a multistep process regulated by an interacting molecular network, and the CDC25 family is important at different steps. However, the expression or function of several other regulators can also be altered in human AML; leukemia-associated alterations of other important regulators will be described below.

### 5.1. Regulation of the G_1_ Phase of the Cell Cycle in Human AML Cells

Cells that have exited mitosis can become quiescent, differentiate or progress to another cell division cycle; initiation of a new cell cycle can be stimulated by the downstream signaling from various growth factor or cytokine receptors and at the same time the transcriptional regulation can be altered to suppress differentiation [98]. However, as summarized in Table 2, the various genetic abnormalities of the AML cells will differ in their effects on various regulators of G_1_/S-phase progression [98,99,100,101,102,103,104,105,106,107,108]. Regulation of the G_1_ phase thus seems to differ between patients according to their AML-associated genetic abnormalities.

CDK2 and CDK4/6 activation in G_1_ can be caused by various molecular mechanisms and not only by CDC25, and several of these molecules are regarded as potential therapeutic targets in human malignancies. The optimal targeting of G_1_ progression is therefore likely to differ between patients, and the possible therapeutic strategies include p53 activation and inhibition of various receptor tyrosine kinases, CXCR4 and CDK4/6 [33,105,107,109,110].

### 5.2. Regulation of the S-Phase 

The regulation of S-phase progression is summarized in Figure 2 and the altered regulation of this phase in human AML cells is described more in detail in Table 3 [49,98,99,102,111,112,113,114,115,116,117,118,119,120,121,122,123,124,125,126,127,128,129]. The regulation of S-phase progression can be altered in primary AML cells, and the alterations can be both upstream and downstream to CDC25. This cell cycle phase is targeted by the antileukemic drugs cytarabine, clofarabine and anthracyclines [130,131,132,133]. Finally, the histone modifier MLL is affected by the various 11q23-involving translocations; MLL functions as a cell cycle regulator through its effects on CDC45 and the function of CDC45 in cell cycle regulation seems to be altered especially in the patient subset with these translocations [102].

### 5.3. Regulation of the G_2_ Phase 

Table 4 gives an overview of important regulators of the G_2_ phase that ends up with CDC25 mediated activation of CDK1/cyclin B; the described abnormalities of G_2_ regulation in primary human AML cells are summarized in the table [95,127,128,129,134,135,136,137,138,139,140]. Similar to the G_1_ phase, the molecular context of CDC25 varies between patients also for this phase, but in contrast to the G_1_ phase where the heterogeneity is mainly due to the direct effects of AML-associated genetic abnormalities, the heterogeneity of G_2_ regulation is mainly determined by differences in expression levels (BRCA1, PP2A, PLK1, cyclin B) or isoform profiles (p53, PP2A).

### 5.4. Regulation of Mitosis 

CDC25 is not an important mediator in regulation of the mitotic step that includes the spindle assembly checkpoint members together with the Aurora kinases and PLK1 [98]. However, several of these regulators can be altered in human AML, the most important being listed below:

**Table 2:** The molecular environment of CDC25—altered regulation of the G_1_ phase in human AML (← stimulation; _┴_ inhibition).

Signaling Cascade		Effects on Cell Cycle Regulation—Effects of Genetic Abnormalities
Receptor ligation or mutations	← RTK	**Receptor ligation and mutations of growth factor receptor tyrosine kinases:** Proliferation of primary AML cells is increased by receptor ligation [103]; receptor tyrosine kinase (RTK) mutations (e.g., c-kit mutations, FLT3-mutations) can cause constitutive signaling [98]. High CXCR4 levels is seen for a minority of patients and is associated with adverse prognosis [105].
↓		**CDK4/6:** These mediators stimulate G_1_ progression and bind/sequester CDK inhibitors (CKI), e.g., p14, p16, p27 [98]. p14 and p27 can show increased expression in AML, p16 may show low expression [106].
STATs		
MAP kinases	↓	**PIM kinases:** These kinases are activated by the upstream receptor ligation together with STAT/MAP kinases and thereafter increase CDK4 activation [98]. They also decrease the effects of the CKIs, and PIM1 activates CDC25 directly (see Table 4).
PLC		
↓		**CKI (CDK inhibitors):** High p27 levels seem to be predictive of complete remission after intensive induction chemotherapy [102].
**CDK4/6**	← PIM kinases	**Phospholipase Cβ 1 (PLC):** The nuclear form of this enzyme is an important checkpoint that controls progression through the G_1_ phase, its activation depends on type 1 insulin-like receptor (IGF-R) and monoallelic deletions seem to be associated with aggressive disease both for patients with AML and patients with preleukemic myelodysplastic syndromes [108].
**Cyclin D**		
↓	_┴_	
	├ CKIs	**CDK2 activation:** The upstream receptor-initiated signaling activates CDK2 through CDK4/6 dephosphorylation.
↓		**Cyclin E:** The expression varies between AML patients, high expression is seen in one third of the patients [99].
**CDK2**	├ p53/p21	**p53 and p21—different p53 isoforms associated with FLT3-ITD and NPM1 mutations:** p53 inhibits G_1_ progression through p2 induction. p53 is mutated only in a minority of AML patients; but the balance between various isoforms differ between patients, e.g., between patients with adverse prognosis FLT3-ITD and NPM1 mutations [107].
**Cyclin E**	← E2F	
	
↓		**E2F:** Inactivation of the Rb gene by CDK4/6 releases the E2F transcription factor that increase transcription of cyclin E; aberrant expression of a variant E2F can be seen in human AML [100,101].
↓		**TET2 mutations:** These loss-of-function mutations are detected in 10-20% of AML patients, loss of TET activity leads to increased proliferation and is associated with adverse prognosis [104].
Entering of **S-phase**		**MLL fusion proteins—11q23 translocations:** The wild-type MLL protein cause histone methylation; its expression normally peaks at the G_1_/S boundary but the fusion protein shows stable expression and therefore seems to abrogate checkpoint control and increase the expression of homeobox transcription factors as described in detail by [98].

**Table 3:** The molecular environment of CDC25 during the S-phase of the cell cycle (← stimulation; _┴_ inhibition).

Signaling Pathway	Additional Regulator	Normal Molecular Function	Abnormality in Human AML
DNA damage Replication checkpoint	Claspin	**ATR:** a serine-threonine kinase activated by DNA damage [98].	**ATR:** stabilizes the chromatin-remodeller MLL (see below), this ability is lost for MLL-fusion proteins encoded by 11q23 translocations [98].
		**Claspin:** activated by ATR through phosphorylation, initiates DNA repair and enhances this DNA damage response through stimulation of CHK1 [120,123].	**Claspin:** differentially expressed in AML cell lines [49]
			**CHK1:** experimental and clinical studies suggest that CHK1 inhibition has antileukemic effects [112,114].
			**Chemotherapy:** cytarabine and clofarabine cause S-phase/replication arrest and CHK1 and CHK2-mediated inhibitory phosphorylation of CDC25 [98].
↓		**CHK1:** a serine/threonine kinase that inhibits CDC25 through addition of inhibitory phosphate groups. Differentially expressed in AML cell lines [111].	
ATR			
↓			
CHK1			
		**Clinical relevance:** MLL-fusion proteins caused by 11q23 translocations can inhibit the MLL-ATR interaction, causing MLL degradation and continued replication [115].	
_┴_			
**CDC25**			
Double-strand break	RAD51C PIM kinase 1 FLT3-ITD	**ATM:** a serine/threonine kinase activated by DNA double strand breaks.	**ATM:** genetic variants of ATM influence treatment outcome, heterozygous ATM 4138C>T is associated with an inferior treatment outcome after intensive chemotherapy [121]. Missense mutations have been detected in AML [118].
		**CHK2:** a serine/threonine kinase that adds inhibitory phosphate to CDC25 and thereby inhibits CDK2.	
Intra-S phase			
Checkpoint			**CHK2:** can be mutated in human AML, but this is uncommon [111].
↓			
ATM		**RAD15C:** Involved in ATM-dependent binding to DNA double-strand breaks required for CHK2 phosphorylation [122].	**RAD51C:** polymorphisms do not influence risk of AML or outcome after chemotherapy [116].
↓			
CHK2			
_┴_			**FLT3-ITD:** this is a common AML mutation associated with chemoresistance and reduced AML-free survival. It shows constitutive signaling and causes activation of CDC25. Normal myeloid differentiation requires activation of the transcription factor C/EBPα; constitutively activated FLT3-ITD causes an inhibitory phosphorylation and thereby a block of differentiation mediated either by ERK1/2 or by CDK1 (see description of the G_2_ phase, Table 4) [127,128,129].
**CDC25**		**PIM kinase 1:** this serine/threonine kinase transduces cytokine-initiated mitogenic signals; it phosphorylates CDC25A directly and this phosphorylation increases the phosphatase activity of CDC25A [126].	
			**Chemotherapy:** topoisomerase inhibitors (etoposide, anthracyclines) favor DNA strand breaks and provoke a checkpoint response involving ATM [98].
**CDC25**		**CDK2:** a serine/threonine kinase activated by cyclin A and cyclin C and inactivated by p21 and p27.	**CDK2:** high activity with fast progression through S-phase has been described in AML [102].
↓			
↓			
CDK2			**Cyclin A:** high activity with fast progression through S-phase has been described [113,117,124].
Cyclin A		**Cyclin A:** regulates S-phase progression, interact with c-Myb [113,117,124].	
Cyclin C			
↓			**Cyclin E:** high activity with fast progression through S-phase has been described in human AML [99].
↓			
**CDC45**		**Cyclin E:** regulates G_1_/S transition	**CDC45:** altered regulation in 11q23 AML [102].
		**CDC45:** regulator of replication, regulated by wild-type MLL [102].	**p21:** deficiency cooperates in t(8;21) variants of AML [119].
			**p27:** downregulated in AML, faster S-phase progression [125].
		**p21:** inhibitor of CDK2 and CDK4	
		**p27:** inhibitor of CDK2 and CDK4	

**Table 4:** The molecular environment of CDC25‒altered regulation of the G_2_ checkpoint in human AML (← stimulation; _┴_ inhibition).

Signaling Cascade		Effects on Cell Cycle Regulation—Effects of AML-Associated Abnormalities
**DNA-damage** ↓ **ATR-ATM** **CHK1-CHK2** _┴_ **CDC25** ↓ **CDK1** **Cyclin B** ↓ **Mitosis**	γ-H2Ax, BRAC1 SET _┴_ ← PP2A ← PLK1 ← FOXM1 ├ p53/p21	**NFκB and cell cycle regulation: **
		Activation of the NFκB system is important for transcriptional regulation, and inhibition of NFκB delays mitotic entry and inhibits transcription of G_2_/M-specific genes, including cyclin B, PLK1, and CDC25B [95]. NFκB is important for AML stem cell functions and their chemosensitivity [96], but it is not known whether its effect on leukemic stem cells is mediated through altered CDC25B expression.
		**The DNA damage response:**
		**γ-H2Ax:** Phosphorylation of this histone is an indication of a DNA damage response, the AML cell level after treatment shows considerable variation between patients [135].
		**BRCA1/2:** DNA repair is closely linked to cell cycle regulation; reduced BRCA1 gene expression is seen for a minority of *de novo* AML but for a majority of secondary AMLs [138].
		**SET:** an endogenous inhibitor of PP2A [134].
		**PP2A:** Inactivation through phosphorylation is seen in primary AML cells for a majority of patients; this inactivation can be caused by either deregulated expression of endogenous PP2A inhibitors (e.g., SET, see above), overexpression of SETBP1 or downregulation of PP2A subunits [134].
		**PLK1:** This kinase is overexpressed in primary AML cells for a majority of patients [137,139].
		**FOXM1:** This transcription factor seems to be overexpressed in primary AML cells for most patients; decreased expression is associated with G_2-_arrest and reduced levels of Aurora kinase B, cyclin B1 and CDC25B together with increased levels of p21 and p27 at the protein level [136].
		**Cyclin B:** Cyclin B1 is commonly expressed in primary AML cells, but the expression pattern (cytoplasmic versus nuclear) varies between patients [140]. The expression is controlled by FOXM1, see above [136].
		p53/p21: See Table 2 and Table 3
		**CDK1 and FLT3 mutations:**
		An alternative pathway for activation of CDK1 is the constitutive activation by FLT3-ITD; this signaling can activate both ERK1/2 and CDK1 and these kinases can then phosphorylate the transcription factor C/EBPα; this phosphorylation will inhibit its transcriptional activity and thereby contribute to the differentiation block in these AML cells [127,128,129].

- *The mitotic checkpoint.* Several regulators of the mitotic checkpoint show decreased levels in primary human AML cells and this weakens the mitotic checkpoint response [141,142,143]. Blinkin can also be a fusion partner with MLL in 11q23 translocations; this fusion molecule seems to cause a dominant negative effect over the wild type protein.
- *Chromatin modeling.* 11q23 translocations resulting in a fusion protein with septin cause disturbed chromatin remodeling [144]. The chromatin-modifying enzyme MLL5 can also be deleted [145,146] thereby altering the mitotic entry.
- *Aurora kinases and PLK1.* The Aurora kinases [147,148,149] as well as PLK1 [137,139,150] can all be overexpressed in primary human AML cells and contribute to disturbances in mitotic entry, progression and/or exit.
- *The t(8;21) abnormality.* This AML-associated abnormality can also disturb this step through downregulation of securin. Furthermore, cyclin B is involved in regulation of mitosis and can be downregulated by the fusion protein encoded by the t(8;21) fusion gene [141].
- *CDK1 and FLT3 mutations.* An alternative pathway for activation of CDK1 is constitutive activation of FLT3-initiated signaling by FLT3-ITD; this signaling can activate both ERK1/2 and CDK1 and these kinases then phosphorylate the transcription factor C/EBPα; this phosphorylation will inhibit its transcriptional activity and thereby contribute to the differentiation block in these AML cells [127,128,129]. Whether this phosphorylation is mediated by ERK1/2 or CDK1 seems to differ between patients [127,151], and this difference between FLT3-ITD patients may depend on the large variation of the ITDs between patients [152]. Thus, the FLT3-ITD abnormalities can alter both differentiation and cell cycle regulation through their effects on CDK1.

### 5.5. Conclusions—Patients are Heterogeneous with Regard to Cell Cycle Regulation of the AML Cells 

The summaries given above and the data presented in Table 2, Table 3 and Table 4 clearly illustrate the patient heterogeneity with regard to cell cycle regulation in their AML cells. This heterogeneity is caused both by direct effects of the AML-associated genetic abnormalities (e.g., fusion proteins), but also by altered intracellular levels of important cell cycle regulators. Thus, the biological context of CDC25 in primary AML cells will differ between patients. These observations further support our hypothesis that the effect of CDC25 inhibition in primary human AML cells will differ between patients, and future biological as well as clinical studies therefore have to address the question whether CDC25 inhibition is effective for most or only a subset of AML patients.

## 6. CDC25 Inhibition in the Treatment of Human AML

### 6.1. CDC25 and the Anticancer Effects of mTOR Inhibition

Inhibition of the PI3K-Akt-mTOR pathway is now considered for the treatment of human AML [89]. However, a recent study described that the mTOR inhibitor rapamycin affected the phosphorylation status of 250 phosphorylation sites in 161 cellular proteins, including several kinases and phosphatases [61]. These authors described that rapamycin-induced activation/phosphorylation of both CDC25B and Akt; silencing of the CDC25 phosphatase through mutation of the phosphorylation site then blocked the activation of Akt in various cancer cell lines. This last observation indicates that phosphorylation of CDC25B is critical for the rapamycin-induced activation of Akt and eIF4E pathways while still suppressing the phosphorylation of the downstream mTOR targets p70S6K and 4E-BPI [153]. The CDC25B-mediated activation of Akt/eIF4E may then represent a resistance mechanism against therapeutic inhibition of mTOR, and combined inhibition of PI3K-Akt-mTOR and CDC25 may then be a strategy to increase the anticancer effect of PI3K-Akt-mTOR inhibition. To the best of our knowledge these effects have only been described for rapamycin, and it is not known whether similar effects are seen for other mTOR inhibitors or for inhibitors targeting other members of this pathway.

Our recent observation that resistance against PI3K/mTOR inhibitors in primary human AML cells was associated with increased mRNA expression of CDC25B also supports the hypothesis that CDC25 inhibition will increase the anticancer effects of PI3K/mTOR inhibition, but at least for AML this combined strategy showed an increased efficiency only for certain patients [89].

To summarize, the PI3K-Akt-mTOR pathway seems to be closely linked to CDC25, especially the CDC25A and CDC25B isoforms in human AML (Table 1), but our present knowledge is fragmentary and cannot explain all the experimental observations. CDC25A seems necessary for Akt-dependent and integrin-initiated proliferation (only examined in a limited number of cell lines), but at the same time signaling through this pathway can inhibit CDC25 through its sequestering in the cytoplasm. On the other hand, high constitutive CDC25B expression has been associated with resistance to PI3K and mTOR inhibitors in primary AML cells. Possible explanations for these apparent discrepancies could be that: (i) PI3K-Akt-mTOR signaling can be modulated by the crosstalk with other pathways; (ii) the final effect of CDC25 in primary human AML cells depends on the CDC25 level; and (iii) the observed effects in a limited number of AML cell lines may not be representative for primary human AML cells in general.

### 6.2. CDC25 and Induction of Differentiation in AML Cells

AML is characterized by accumulation of immature leukemia cells; these cells have a block in their differentiation but for some patients morphological signs of differentiation can be seen [154]. The function of CDC25 also seems to be affected by AML cell differentiation, *i.e.*, to differ between AML cells with no signs of differentiation and cells showing monocytic differentiation [49]. Similarly, CDC25 is downregulated when the monocytic AML cell line ML-1 is stimulated to differentiate towards granulocytes *in vitro* [155]. However, it is not known whether the limited signs of differentiation seen for primary AML cells will have any major impact on the level of CDC25 or the effects of CDC25 inhibition *in vivo*.

## 7. The Possible Use of CDC25 Inhibitors in the Treatment of human AML

### 7.1. AML is a Heterogeneous Disease—the Possible Use of CDC25 Inhibitors Only in Subsets of Patients

AML patients are classified according to the recently published WHO classification [154], and this classification is based on differences in morphology, predisposition (AML secondary to myelodysplastic syndromes (MDS) or chemotherapy), and the detection of defined genetic abnormalities. Even by using only this limited number of parameters it is possible to classify the patients into a wide range of subsets that only reflects a part of the biological heterogeneity in human AML. Firstly, even though AML is generally defined as a disease with at least 20% leukemic blasts among bone marrow nucleated cells, there are exceptions from this definition, the most important being that patients with certain low-risk genetic abnormalities (*i.e.*, inv(16), t(16;16), t(8;21)) are defined to have AML even when the number of blasts is <20%. Secondly, the age of the patient seems to reflect biological differences independent of the WHO classification. Thirdly, both the cytogenetic and molecular genetic abnormalities show a much more extensive heterogeneity than reflected in the WHO classification. Finally, a large number of differences at the protein level has also been detected [107,156,157]. In our opinion it would not be surprising if CDC25 inhibitors would be effective only for a subset of these highly heterogeneous patients. Future studies should therefore try to identify biomarkers for susceptibility to CDC25 inhibition.

### 7.2. Combined Targeting of Cell Cycle Regulation—the Possible Combination of CDC25 Inhibition with Inhibitors of PLKs, AURORA Kinases or CDK Inhibitors

Targeting of cell cycle regulation is already considered for the treatment of human AML, and especially inhibition of Aurora kinases or PLK1 has been studied in clinical trials as described in detail in a recent review [139]. These clinical studies have included hundreds of patients and both specific and multikinase inhibitors have been tried. The inhibitors are effective and partial responses or stable disease have been observed even for patients with advanced or multiresistant AML. Both PLK1 and Aurora B interact and activate CDC25 (Figure 3), and the clinical experience with these alternative cell cycle inhibitors suggests that CDC25 inhibition is effective in human AML.

The 11q23 chromosomal abnormalities involving the H3K4 methyltransferase MLL give rise to an aggressive subtype of AML [154]. A recent study described that AML cells driven by MLL-AF9 derived from t(9;11) are exceptionally reliant on CDK6 but not its functional homolog CDK4, and that the growth inhibition induced by CDK6 depletion is mediated through enhanced myeloid differentiation [158]. CDK6 seems to have a similar function also for other translocations involving 11q23. The same seems to be true in acute lymphoblastic leukemia with 11q23 fusion genes [159]. CDK6 inhibition by the combined CDK4/6-inhibitor PD0332991 seems to have similar effects [159]. CDC25 is a regulator of the CDK4/CDK6/cyclin D complex (Figure 2), and the possible use of CDC25 inhibitors alone or in combination with CDK targeting treatment should therefore be further explored.

The sulfonamide derivative E7070/indisulam [160,161,162] inhibits carbonic anhydrases I, II, IV and IX [160]. This agent was originally identified as an antitumor agent due to its inhibitory effects on cell cycle progression in human cancer cell lines, including (i) suppression of cyclin E expression; (ii) reduced protein expression of cyclin A, cyclin B, CDK1 and CDK2; (iii) inhibition of CDK2 and Rb phosphorylation; and (iv) induction of p53 and p21 [160]. E7070 thereby disrupted cell cycle progression at different steps including G_1_/S and G_2_/M transitions. Combination of E7070 and CDC25 inhibition would thus represent an extensive inhibition of several steps in the cell cycle together with inhibition of adaptation to the hypoxic bone marrow microenvironment of human AML [163,164,165].

E7070 has been investigated in a recently reported clinical trial [166]. The study included 20 patients with advanced or chemoresistant AML; patients were initially treated with E7070 alone and, if no response, they received E7070 plus idarubicin and cytarabine. No patient responded to E7070 alone, but eight patients achieved hematological remission after combination therapy. Two patients had prolonged marrow hypoplasia exceeding 42 days, the only grade ≥3 nonhematological toxicity was hyperbilirubinemia. The results are encouraging, but this was only an interim analysis and one has to await the final report.

### 7.3. Combination of CDC25 Inhibitors with Conventional Chemotherapy

PLK/Aurora kinase inhibitors seem to enhance the antileukemic effect of several cytotoxic drugs used in the clinical treatment of human AML, including cytarabine, daunorubicin and etoposide [139]. Furthermore, many cell cycle inhibitors show minimal cross-resistance with conventional anticancer agents [139]. This may also be true for CDC25 inhibitors, and the possibility to combine CDC25 inhibitors with conventional antileukemic drugs should be further investigated in experimental studies and, eventually, in later clinical studies.

One possibility would be to combine CDC25 inhibitors with intensive chemotherapy to increase the antileukemic effect and thereby improve the long-term AML-free survival. However, an alternative strategy could be to combine CDC25 inhibitors with low-toxicity disease-stabilizing strategies in patients that are unfit for the intensive and potentially curative treatment. If this last strategy is considered, CDC25 inhibitors may be combined with all-*trans*-retinoic acid (ATRA) plus valproic acid [167], low-dose cytarabine [168], hydroxyurea and/or 6-mercaptopurine [167,168,169,170,171].

### 7.4. Toxicity versus Antileukemic Efficiency

Several side effects have been observed with other pharmacological agents targeting cell cycle regulation [139]. Hematological toxicity with neutropenia (eventually febrile neutropenia) and thrombocytopenia seems to be relatively common for Aurora kinase and PLK1 inhibitors. The preliminary experience also for other cell cycle inhibitors including ARQ-501 [81,82] is that anemia and eventually other signs of bone marrow toxicity are relatively common. Combination of different cell cycle-targeting strategies or cell cycle targeting together with intensive chemotherapy will possibly lead to more severe cytopenias. This problem may be less severe if CDC25 inhibition is tried as a part of the post-remission/consolidation treatment when disease-induced bone marrow failure is not present. Gastrointestinal toxicity due to severe mucositis is another severe problem in conventional AML treatment and such toxicity is also common and can even be dose-limiting for PLK/Aurora kinase inhibitors [139]. Gastrointestinal toxicity may also become a problem if conventional AML chemotherapy is combined with CDC25 inhibition. Thus, the toxicity has to be carefully evaluated if CDC25 inhibition is combined either with other cell cycle-targeting strategies or with conventional chemotherapy, even for low-dose disease-stabilizing chemotherapy.

### 7.5. Inhibition of Several Therapeutic Targets through the Use of a Single CDC25 Inhibitor

Most CDC25 inhibitors inhibit all three CDC25 isoforms, but the relative strength of inhibition for each of the three isoforms varies between inhibitory agents [5]. Further studies are needed to clarify whether any of the three isoforms is of particular biological importance in human AML.

CDC25 has its own network of interacting kinases (Table 5) [21,25,29,30,32,33,44,47,48,50,60,61,62,63,64,65,172]. Its activity is modulated by various kinases and at the same time CDC25 regulates the activity of several kinases. CDC25 inhibition thereby offers the possibility to target several intracellular regulators through inhibition of a single molecule. As described above, CDC25 even seems to contribute to the regulation of cytoplasmic signaling pathways. Only future studies can clarify whether this represents a possibility for enhanced antileukemic activity rather than a risk of severe toxicity, and studies in AML animal models will then be important [173,174].

**Table 5:** CDC25 and the most important members of its interacting kinase network—an overview of kinases that activate CDC25 and kinases that are activated by CDC25 dephosphorylation.

Upstream Events Affecting CDC25 Activation (See Also Figure 3)	
Akt/PKB [60,62,63,64,65]	This pathway can be an upstream inhibitor of CDC25 through sequestering it in the cytoplasm, but at the same time CDC25A is necessary for Akt-initiated proliferation.
MAP kinases [30]	Negative regulator during cellular stress.
CDK1/cyclin B [44]	Activates CDC25B and CDC25C in a positive feedback loop.
CDK2/cyclin E [21,29]	Activates CDC25A in a positive feedback loop.
PLK1 [44]	Activates CDC25 and promotes mitosis; both direct and indirect activating effects.
Aurora kinases [47,48]	Activate CDC25s and promote mitosis; both direct and indirect activating effects via activation of PLK1.
Wee1 and Myt1 kinases [33]	Inhibition of CDC25s.
CHK1 [50,172]	Inactivates all three isoforms of CDC25; it also inhibits CDC25s indirectly, e.g., through PLK1 inhibition.
CHK2 [50,172]	Inactivates all three isoforms of CDC25; it also inhibits CDC25s indirectly, e.g., through PLK1 inhibition.
CDK1 [30,32]	CDK1/cyclin A is dephosphorylated by CDC25B in late S-phase. Dephosphorylation of CDK1/cyclin B by CDC25 is a rate-limiting step for transition from G_2_ to mitosis.
CDK2 [25,30]	Activated during several steps in the cell cycle. Associates with cyclin E during G_1_/S transition and cyclin A during S-phase.
CDK4 [25]	Important for entry into S phase. Associates with cyclin D.
CDK6 [25]	Important for entry into S phase. Associates with cyclin D.
Akt/protein kinase B [61]	Its phosphorylation can be regulated by CDC25B.

## 8. Final Comments

As outlined above, CDC25 inhibition can be achieved directly and indirectly through inhibition of its upstream signaling pathways or its interacting molecules. The direct inhibitors have not been investigated in clinical trials (or are in the early stages of clinical trials) and their pharmacokinetic properties have to be better characterized, but despite this they should be regarded as promising therapeutic agents in human AML because they offer the possibility to target several proteins, including various CDC25 isoforms as well as several kinases, through the administration of single pharmacological agents. Their possible use in AML treatment either as single drugs or in combination with other drugs should therefore be further investigated. The first step will then be a further characterization of the effects at the cellular level and especially in primary human AML cells and this should be followed by studies in AML animal models [173,174] as a basis for the design of further clinical studies.
